# Design of an automated robotic microinjection system for batch injection of zebrafish embryos and larvae

**DOI:** 10.1038/s41378-023-00645-6

**Published:** 2024-01-29

**Authors:** Zhongyi Guo, Nana Ai, Wei Ge, Qingsong Xu

**Affiliations:** 1 Department of Electromechanical Engineering, Faculty of Science and Technology, University of Macau, Macau, China; 2 Department of Biomedical Sciences and Centre of Reproduction, Development and Aging (CRDA), Faculty of Health Sciences, University of Macau, Macau, China

**Keywords:** Electrical and electronic engineering, Microengraving

## Abstract

This paper presents the design of a vision-based automated robotic microinjection system for batch injection of both zebrafish embryos and larvae. A novel visual recognition algorithm based on an automatic threshold and excessive dilatation is introduced to accurately identify the center of zebrafish embryos and larval yolks. A corresponding software system is developed using the producer-consumer model as the framework structure, and a friendly user interface is designed to allow operators to choose from a range of desired functions according to their different needs. In addition, a novel microstructural agarose device is designed and fabricated to simultaneously immobilize mixed batches of embryos and larvae. Moreover, a prototype microinjection system is fabricated by integrating hardware devices with visual algorithms. An experimental study is conducted to verify the performance of the robotic microinjection system. The results show that the reported system can accurately identify zebrafish embryos and larvae and efficiently complete batch microinjection tasks of the mixtures with an injection success rate of 92.05% in 13.88 s per sample. Compared with manual and existing microinjection systems, the proposed system demonstrates the merits of versatility, excellent efficiency, high success rate, high survival rate, and sufficient stability.

## Introduction

With the rapid development of micro/nanotechnology tools, numerous micromanipulators, including microarrays^1,2^, microfluidic chips^3,4^, microinjectors^5,6^, and microgrippers^7–9^, are being widely utilized in biomedical devices^10,11^, material science^12–14^, and microelectromechanical systems^15,16^. As an efficient method of transporting external substances into cells, microinjection plays a crucial role in biological experiments. Zebrafish are regarded as a powerful model organism^17,18^. The zebrafish genome has been fully sequenced, and it has 74.1% homology with humans^19,20^. Based on this similarity, zebrafish have been studied against various human diseases, such as Noonan syndrome, osteoporosis, atrial fibrillation, autism spectrum disorders, and leukemia. Compared to mice with a production cycle of approximately 3 weeks and an average of 6–8 offspring per pregnancy, zebrafish have the advantages of high fecundity and high embryo yield, which can significantly shorten the experimental period and effectively reduce the deviation in experimental results caused by individual differences in the population of animal research subjects. Zebrafish also offer other experimental advantages, such as transparent embryos, easy breeding, rapid development, external fertilization, in vitro development, and multiple organs with regenerative capacity, which have been extensively used in studies of genetics^21^, developmental biology^22^, neurobiology^23^, oncology^24^, regeneration and stem cell research^25^, disease models and drug screening^26^, cellular biophysical analysis^27–29^ and more.

Therefore, the development of microinjection technology for zebrafish can profoundly impact biomedical research. A widely used microinjection method, manual microinjection has low efficiency and poor stability limitations. It is also time-consuming and costly to train skilled operators. Especially considering the demands of high-intensity and high-repeatability batch microinjection, the above limitations are severe. Therefore, developing a robotic microinjection system for zebrafish samples is essential to improving the injection efficiency and success rate and further liberating human operators.

Certain studies have made significant advances in autonomous microinjection in zebrafish embryos^30^. Xie et al.^31^ presented a robot-assisted microinjection system for zebrafish, but their system could complete only a single sample microinjection in one task and was not suitable for large-scale microinjection tasks. To reduce puncture force and thereby reduce physical damage to embryos, Shang et al.^32^. proposed a 7-DOF rotation thrust microrobotic system, which was also not suitable for batch microinjection tasks. Distinct from these studies, Wang et al.^33,34^ presented a fully automated robotic system for microinjection of batch zebrafish embryos at a speed of 15 zebrafish embryos per minute. However, their system was not applicable to already developed zebrafish larvae, which limited the application scenarios of automatic microinjection systems. In addition, Huang et al.^35^, Lu et al.^36^, and Feng et al.^37^. researched force feedback systems for batch injection of zebrafish embryos, yet these systems were also unable to complete the microinjection of zebrafish larvae.

Developing a robotic microinjection system for zebrafish larvae is more challenging than for zebrafish embryos because the larvae are well-developed organisms with more complex internal and external structures. Their developing organs make it more difficult for injection tools to identify and locate target sites. In addition, the larvae already have vitality and may move to change their position under the stimuli induced by needles^38^. Nevertheless, with the development of robotics and machine vision technology in recent years, significant achievements have been made in automatic microinjection research for zebrafish larvae. Zhuang et al.^39^, Qian et al.^40^, and Zhang et al.^41^. have all achieved automatic microinjection for zebrafish hearts. Zhang et al.^42^. also presented an integrated microfluidic system for zebrafish larval heart microinjection with a cardiac injection success rate of 94%; however, the average microinjection time of one sample was 75 s. Significantly, the current studies have achieved sufficient injection accuracy in zebrafish larvae at the expense of time, which is inadequate when facing high-throughput requirements. Particularly in some disease models and drug screening studies, both zebrafish batch embryos and batch zebrafish larvae need to be injected with drugs to observe their effects. Regrettably, no research to date has successfully implemented an integrated system capable of autonomously conducting batch microinjection of zebrafish embryos and larvae.

Recognition algorithms for zebrafish embryos and larvae have made significant progress. Some efforts use traditional visual processing methods to identify the microinjection target^43,44^, such as binarization, template matching, and contour extraction. Nevertheless, these methods are not sufficiently robust in terms of anti-interference, especially in environments containing miscellaneous matter. With the strong and recent development of deep learning techniques, convolutional neural network technology has been applied to automatic microinjection systems for target object recognition^45,46^. After extensive model training, such a system can provide sufficiently high recognition accuracy. However, the convolution operation in the early training phase for datasets and the program execution phase for target object recognition further increases the time consumption. Therefore, it is necessary to develop an efficient and accurate visual recognition algorithm that can be used to simultaneously identify the injection points of zebrafish embryos and larvae and achieve high-throughput automatic robotic microinjection. In addition, the currently developed microinjection system lacks a friendly user interface (UI), making it difficult for biological experimenters to operate without engineering experience. Therefore, developing a complete operating system similar to commercial software with a friendly UI is crucial to facilitate users’ operation and developers’ further development.

Here, research is presented to address these existing challenges. In general, the automatic microinjection system developed in this study encompasses four main innovations. First, a novel vision recognition algorithm is implemented based on an automatic threshold and excessive dilatation to quickly and effectively identify the centers of zebrafish embryos and larval yolks for the first time. Second, a software system for automatic microinjection is constructed based on the producer-consumer framework model, and a friendly UI is specifically designed, greatly facilitating biomedical researchers. Third, a microstructural agarose medium (MAM) is designed to address the issue of batch sample fixation of zebrafish embryos and larvae simultaneously. Finally, a complete prototype system is developed, featuring a novel and efficient automatic microinjection workflow to realize high-throughput microinjection. Through experimental studies, the effectiveness of the designed software and hardware system is verified for use in automatic batch microinjection of zebrafish embryos and larvae.

The remainder of this paper is structured as follows. First, the implementation process of the proposed recognition algorithm is described. Then, a detailed introduction to the software and hardware system composition is provided. Next, experimental studies are performed to assess and validate the performance of the robot microinjection system. Finally, a discussion and summary are presented, and relevant materials and methods are provided.

### Machine vision algorithm development

**Fig. 1:**
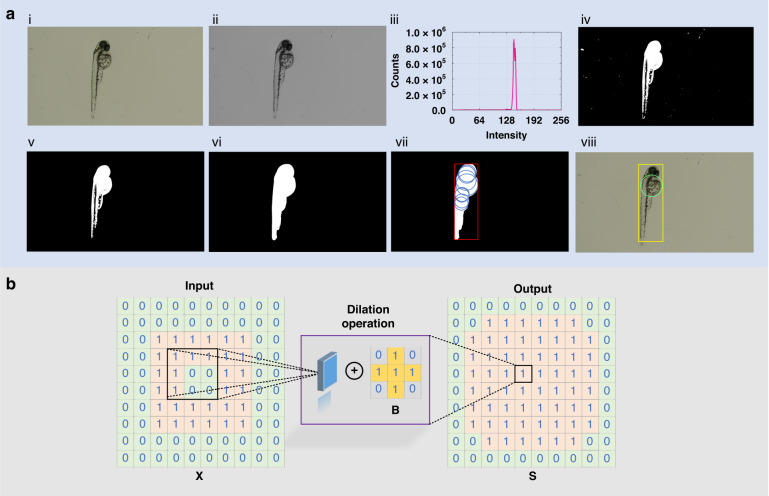
**Implementation process of the visual recognition algorithm.** **a** Recognition process of the yolk center in one zebrafish larva. **b** Schematic diagram of the image dilation principle

Image graying is typically adopted as a preprocessing step in image processing to prepare for subsequent upper-level operations such as image segmentation, image analysis, and target recognition. There are multiple methods to achieve image graying, and the mean value method, which can obtain a relatively soft grayscale image, is adopted here. As shown below, the calculation process averages the three components in an RGB color image to obtain a grayscale value:

1 $$Gray(i,j)=\frac{(R(i,j)+G(i,j)+B(i,j))}{3}$$

where *Gray* is the obtained grayscale value, (*i*, *j*) denotes the pixel, and *R*, *G*, and *B* represent red, green, and blue components in the color image, respectively. Figure 1a-i shows the color image of zebrafish larvae obtained by a camera, and the grayscale image is obtained by the mean value method, as illustrated in Fig. 1a-ii.

Then, the number of pixel points corresponding to each grayscale value is obtained by the histogram function, as shown in Fig. 1a-iii. The horizontal axis in this figure represents the grayscale value, and the vertical axis represents the number of pixels. Global grayscale thresholding is then performed on the grayscale image. In this process, a grayscale threshold *K* needs to first be set. The pixels with grayscale values less than or equal to the threshold *K* are considered background, and the pixel value is set to 0. The pixels with grayscale values greater than the threshold *K* are regarded as foreground, and their pixel value is set to 1, thus completing binary image processing^47^. The grayscale threshold *K* can be determined through manual adjustment via trial and error to obtain the optimal threshold; however, this method is not robust. Hence, the threshold determination method based on maximum entropy is designed to determine the threshold value automatically.

Information entropy is a concept that is used to measure the amount of information. Similar to entropy in physics, the more uniform the distribution is, the greater the entropy. As mentioned, the grayscale threshold divides all pixels in an image into two categories: foreground and background. When the pixels in each category are evenly distributed, it indicates that the entropy is at its maximum. Due to the system entropy accumulation, the entropy of the entire image is at its maximum. Therefore, this principle can be used to determine the optimal threshold *K*, and the specific implementation process is given as follows:

2 $${p}_{i}=\frac{h(i)}{\mathop{\sum }\limits_{i=0}^{N-1}h(i)}$$

where *p*_*i*_ represents the event probability of the grayscale value *i*, *h* (*i*) denotes the number of pixels for each grayscale value in the image, corresponding to the *y*-axis coordinates in Fig. 1a-iii, and *N* denotes all grayscale values in the image (*N* = 256 for an 8-bit grayscale image).

If *k* is the grayscale threshold, the following formula can be obtained:

3 $${P}_{0}=\mathop{\sum }\limits_{i=0}^{k}{p}_{i}$$

4 $${P}_{1}=\mathop{\sum }\limits_{i=k+1}^{N-1}{p}_{i}=1-{P}_{0}$$

where *P*_0_ denotes the probability of a grayscale value between 0 and *k*, and *P*_1_ represents the probability of a grayscale value between *k* + 1 and *N*−1.

The expression of information entropy *H* is defined below:

5 $$H=-\mathop{\sum }\limits_{i=0}^{N-1}p(i)\mathrm{ln}\,p(i)$$

After setting the grayscale threshold, the entropy of an image consists of two parts: the entropy of the background (*H*_0_) and the entropy of the foreground (*H*_1_). Inserting (3) and (4) into (5) yields:

6 $${H}_{0}=-\mathop{\sum }\limits_{i=0}^{k}\frac{{p}_{i}}{{P}_{0}}\mathrm{ln}\,\frac{{p}_{i}}{{P}_{0}}$$

7 $${H}_{1}=-\mathop{\sum }\limits_{i=k+1}^{N-1}\frac{{p}_{i}}{{P}_{1}}\,\mathrm{ln}\frac{{p}_{i}}{{P}_{1}}=-\mathop{\sum }\limits_{i=k+1}^{N-1}\frac{{p}_{i}}{1-{P}_{0}}\mathrm{ln}\,\frac{{p}_{i}}{1-{P}_{0}}$$

Therefore, the entropy of the entire image can thus be expressed as:

8 $${H}_{{\rm{sum}}}={H}_{0}+{H}_{1}=-\mathop{\sum }\limits_{i=0}^{k}\frac{{p}_{i}}{{P}_{0}}\mathrm{ln}\,\frac{{p}_{i}}{{P}_{0}}-\mathop{\sum }\limits_{i=k+1}^{N-1}\frac{{p}_{i}}{1-{P}_{0}}\mathrm{ln}\,\frac{{p}_{i}}{1-{P}_{0}}$$

Furthermore, the optimal grayscale threshold *K* can be obtained as follows:

9 $$K=\text{arg}\,\max {H}_{{\rm{sum}}}(k),\,0\le k\le N-1$$

Then Fig. 1a-ii is operated using the abovementioned maximum entropy-based automatic threshold method, and the resulting binary image is shown in Fig. 1a-iv. Because impurities may exist in the Petri dish, noise information will appear on binary images. Therefore, advanced morphological functions are designed to achieve low-pass filtering. The resulting filtered image is shown in Fig. 1a-v. Comparing Fig. 1a-iv, the method’s filtering effect is evident and shown to be capable of providing high-quality images for further target recognition. Due to the transparent body of zebrafish, part of the body information has been lost and processed as background (black), as shown in Fig. 1a-v, which is not convenient for subsequent visual recognition. Therefore, a dilation function is innovatively introduced to further process the image of zebrafish.

For a given pixel point *A*_0_ in an image, after using the dilation function, the value of *A*_0_ will become the maximum value in the neighborhood corresponding to the structural element:

10 $${A}_{0}=\,\max ({A}_{i})$$

where *A*_*i*_ is the pixel value in the neighborhood corresponding to the structural element.

For a binary image, a dilation function can be used to expand the boundary points of the target object. The background points in contact with the object can be merged into the object, and the holes after image segmentation can be filled. Therefore, this method can effectively solve the problem that the zebrafish body cannot be recognized due to transparency. The specific implementation process is given as follows:

11 $${\bf{S}}={\bf{X}}\oplus {\bf{B}}=\{x,y|{{\bf{B}}}_{xy}\cap {\bf{X}}\,\ne\, \varnothing \}$$

where **X** is the original image matrix, **B** represents the structural element matrix, and **S** describes the new output image matrix. As shown in Fig. 1b, the image dilation process operates similarly to a convolution process, where the structural element matrix **B** moves sequentially on the original image matrix **X**. During the movement process, the maximum value of the elements covered by **B** is used to replace the value at the center position of **B**. Each iteration completes one dilation process.

Notably, image dilation does not change the image contour shape. This result indicates that if the outer contour is a circle, the position coordinates of the circle’s center before and after dilation do not change. This performance is vital for identifying the position of the center of zebrafish embryos and larval yolks. Based on this trend, to avoid losing as much information as possible, we adopt the multiple dilation method, called excessive dilation. Specifically, when processing the zebrafish binary image, we set the size of the structure element matrix to 9 × 9 and perform multiple iterations, with the number of iterations set to 11 to achieve excessive dilation. Figure 1a-vi shows the binarization diagram of the zebrafish larva after excessive dilation.

After excessive dilation, we conducted particle analysis based on the binary image, outputting all regions of interest and labeling them with red rectangular wireframes. Then, we performed circle detection on the image and provided the recognized circles. The recognition result is shown in the blue circular wireframes in Fig. 1a-vii. Considering the body structure of 2-dfp zebrafish larvae, the center of the largest circle in diameter is selected as the output target, i.e., the focus is on the center of a zebrafish larval yolk, as shown in the green circular wireframe in Fig. 1a-viii. By comparing the area sizes of all regions of interest, only the region with the most significant area is considered the final region of interest, as captured by the yellow rectangular box in Fig. 1a-viii.

**Fig. 2:**
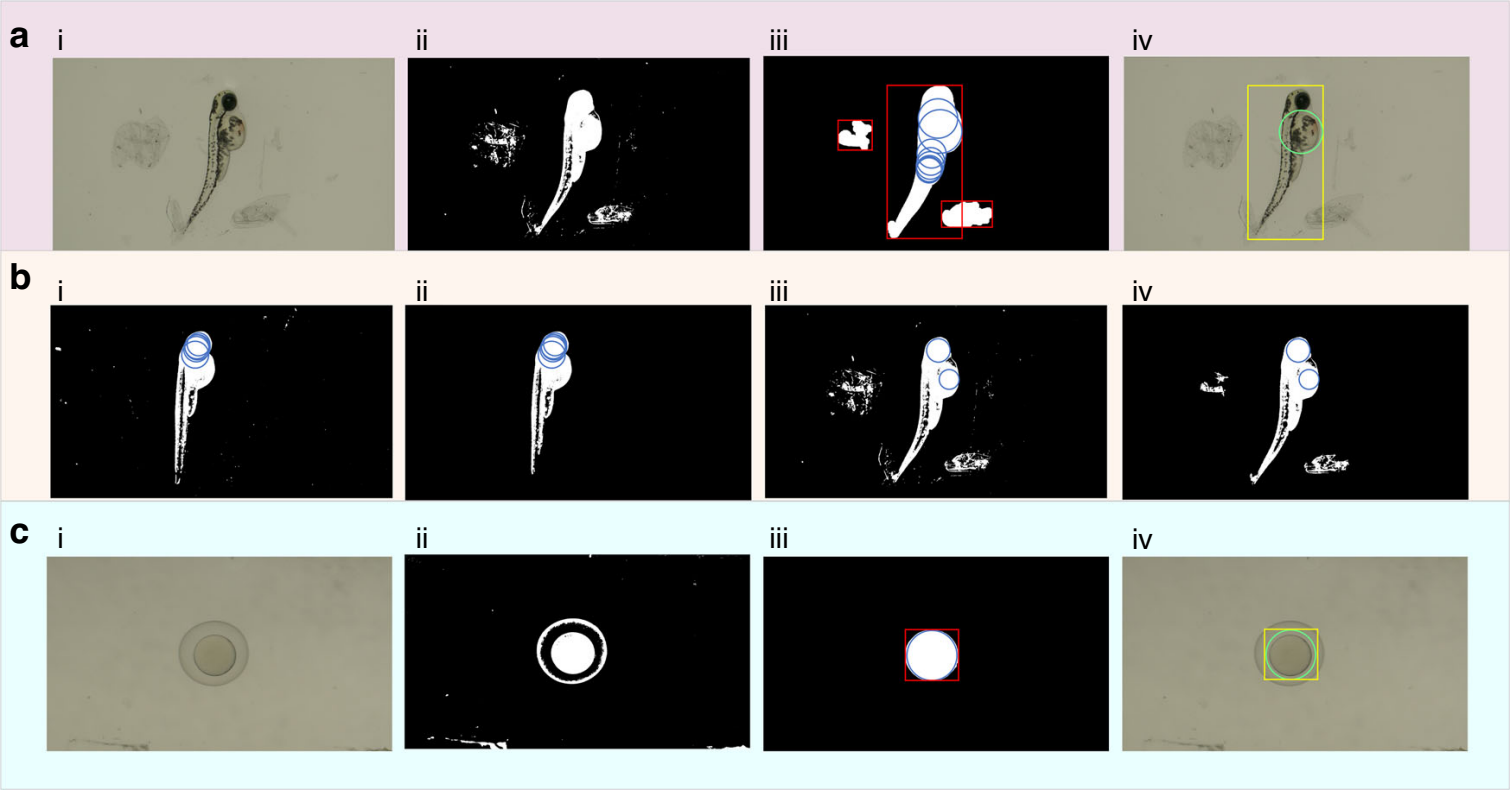
**Verification of algorithm functions.** **a** Verification of the algorithm’s anti-interference ability. **b** Verification of the necessity of the excessive dilation algorithm. **c** Verification of the recognition process to the zebrafish embryo’s center

To demonstrate the anti-interference ability of the algorithm to the impurities, we identified the zebrafish larvae with obvious impurity information in the field of vision, as shown in Fig. 2a. The algorithm recognized the impurity information and only output the region of interest where the zebrafish larva was located, thereby accurately providing the location information of the yolk center.

To demonstrate the necessity of the excessive dilation algorithm, we removed the excessive dilation in the visual algorithm and performed visual recognition of Fig. 1a-i and Fig. 2a-i. The results indicate that even if the optimal threshold is automatically obtained through the maximum entropy method, the central position information of the yolk cannot be recognized regardless of whether filtering is performed, as shown in Fig. 1b.

Compared with zebrafish larvae, zebrafish embryos are similar to circles with more regular shapes, making visual recognition easier. The proposed algorithm is used to detect the central position of zebrafish embryos. A complete detection flowchart is shown in Fig. 1c. The algorithm accurately gave the outline information (yellow rectangular wireframe) and accurate central position information (green circular wireframe) of the zebrafish embryo. In other words, the algorithm can be simultaneously applied to efficiently locate and recognize the center of the zebrafish larval yolk and the center of the zebrafish embryo.

### Prototype development of the automatic microinjection system

#### Software system development

**Fig. 3:**
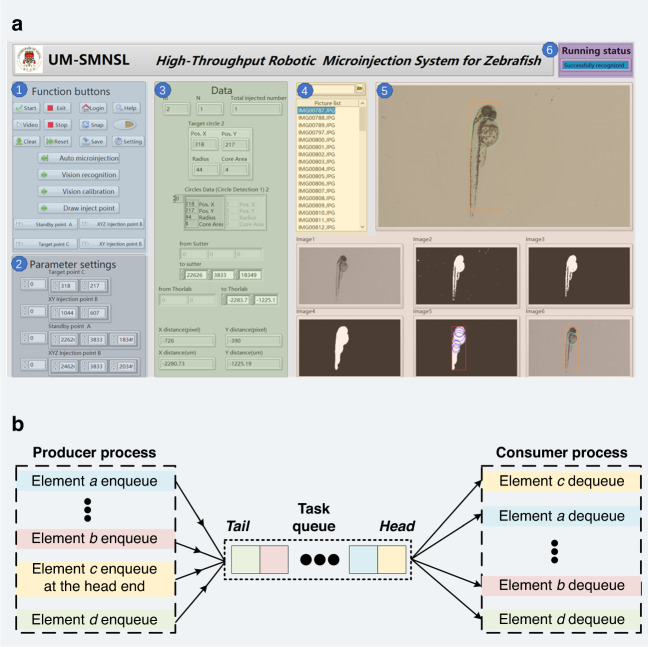
**Developed software system.** **a** Design of the user interface. **b** Schematic diagram of the producer-consumer model

An independent software system for automatic batch microinjection of zebrafish embryos and larvae was developed based on LabVIEW. The software system provides functions such as initialization, visual calibration, visual recognition, and motion control. A user-friendly UI is designed for the software system, as shown in Fig. 3a. The UI comprises six different regions: the function button region, the parameter setting button region, the data display region, the picture list region, the image display region, and the running status region. First, a function button region in the upper-left corner (i.e., region 1 in Fig. 3a) includes system function buttons, visual processing function buttons, and motion control function buttons. In this region, users can choose to perform different functions based on different needs. The system function buttons include functions such as start, exit, login, and help. Notably, if a user clicks the login button and enters the wrong username and password, the other essential buttons on this UI will be disabled. The visual processing function buttons include video, snap, visual calibration, and visual recognition. The visual algorithm is designed for zebrafish embryos and larvae based on an automatic threshold and excessive dilation, which is integrated into the visual recognition function buttons. The UI also retains a parameter setting button region (corresponding to region 2 in Fig. 3a), which allows users to change parameter settings according to different operational tasks to achieve optimal results. Certain key data during software operation are placed in the data display region (corresponding to region 3 in Fig. 3a), where users can obtain meaningful and detailed data. The picture list region (corresponding to region 4 in Fig. 3a) in the middle of the UI allows operators to load images obtained from different folders. Region 5 is the image display region. The largest image display window is the video display region, which can obtain image information and record the microinjection process in real time. The six image windows in the bottom right corner provide users with a more intuitive display of the image processing process for the proposed visual algorithm. Additionally, the running status region in the upper-right corner (corresponding to region 6 in Fig. 3a) displays the real-time program running status, which facilitates users in locating problems quickly when the program reports an error.

The system framework in the software is based on the producer-consumer model. This model refers to communicating between producers and consumers through a buffer (usually a queue). After the producers produce data, they do not need to wait for consumers to process it. Instead, they directly place it in a buffer. Consumers do not seek producer data and instead retrieve it directly from the buffer. This architecture maintains concurrent processing between producers and consumers, balances their processing capabilities, and reduces coupling between producers and consumers. A schematic diagram of its working principle is shown in Fig. 3b. When the user selects different functions to be executed (corresponding to different function buttons in the UI), the elements *a*, …, *b*, *c*, and *d* sequentially enter the task queue and wait for the corresponding program to execute in the consumer process. Notably, because element *c* is enqueued at the head end, it is performed first, and the remaining tasks are executed in the order given by the task queue. This system framework effectively saves program runtime, avoids task conflicts, and provides convenience for the subsequent dilation of more functions.

### Hardware system development

In the microinjection process, to enable the microneedle to complete the puncture process accurately and avoid surface sliding, it is necessary to immobilize the target zebrafish embryo or larva. Standard immobilization methods include vacuum adsorption and microchannels. Since the above process can immobilize only one target object at a time, it is unsuitable for high-throughput microinjection. Hence, this paper designed a novel MAM to immobilize batch zebrafish embryos and larvae, which allows subsequent highly efficient microinjection.

**Fig. 4:**
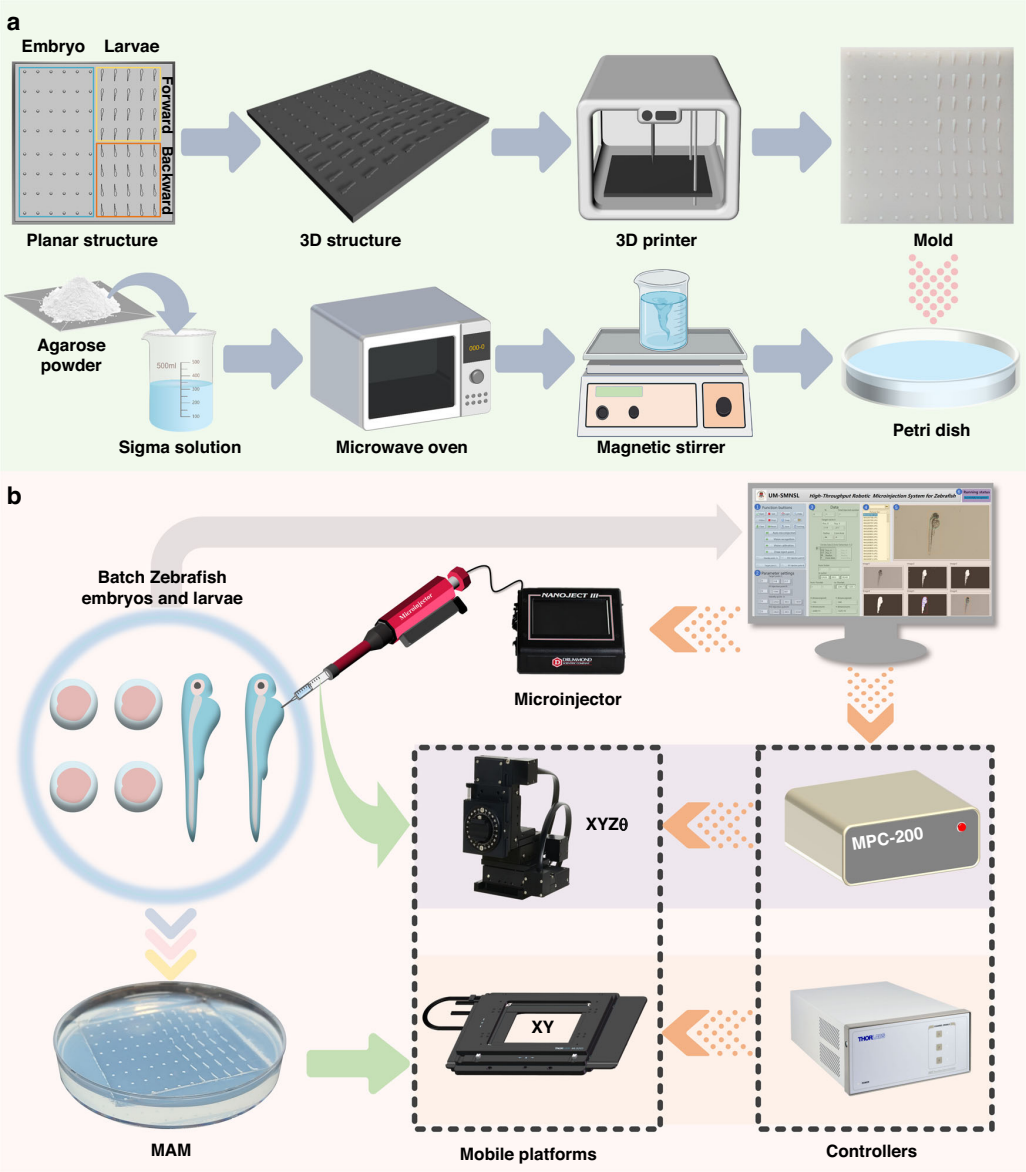
**Development of the automatic microinjection system.** **a** Fabrication of the MAM for immobilizing batch zebrafish embryos and larvae. **b** Hardware device and information communication process in the automatic microinjection system

First, a new mold with protrusions is designed. Its planar structure consists of three components, as shown in Fig. 4a. The blue wireframe on the left of the planar structure is the microarray mold for batch immobilization of zebrafish embryos; the right side is the microarray mold for zebrafish larvae. To avoid overturning when placing zebrafish larvae, it is specifically divided into forward and backward areas according to larval head orientation. The yellow wireframe in the upper-right corner of the planar structure is the forward area (head up), and the orange in the lower right corner is the backward area (head down). Figure 4a also illustrates the mold’s three-dimensional (3D) structure more intuitively. Then, the mold is fabricated using a 3D printer. Finally, we use this mold to manufacture the MAM. The specific materials used and the production process are detailed in the Materials and Methods section.

Table 1 is a detailed introduction to the main hardware equipment of the system. The developed prototype of an automatic microinjection system for batch zebrafish embryos and larvae is shown in Fig. 4b, and it clearly illustrates the information communication process between the hardware and software systems and different devices. Specifically, batches of zebrafish embryos and larvae are placed on the customized MAM under the microscope. The visual recognition algorithm integrated into the developed software system can identify zebrafish embryos and larvae. After the user clicks the *Auto microinjection* button in the UI, the system recognizes the target object. Then, control signals are sent to the controller (BBD-103) through the personal computer, and the XY platform (MLS-203) moves the target object on the MAM to the injection position. When executing microinjection, a set of control signals are sent to the controller (MPC-200) through the computer, and the XYZθ platform (MPC-385) carries the microinjector (Nanoject III) to puncture the target object at the injection position.

**Table 1:** Hardware equipment of the system

No.	Equipment	Mode	Manufacturer	Function
1	Microscope	SZ61	Olympus Inc.	Obtain real-time images
2	XY platform	MLS-203	Thorlabs Inc.	Carry the MAM for directional movement
3	DC servo controller of XY platform	BBD-103	Thorlabs Inc.	Control of the XY platform
4	Joystick of XY platform	MJC-001	Thorlabs Inc.	Manual remote Control of the XY platform
5	Microinjector	Nanoject III	Drummond Scientific Corp.	Complete the task of injecting drugs
6	Microneedle	Glass Capillaries 4878	World Precision Inc.	Complete puncture
7	XYZθ platform	MPC-385	Sutter Inc.	Achieve the movement of microinjector.
8	Controller of XYZθ platform	MPC-200	Sutter Inc.	Control the XYZθ platform
9	Joystick of XYZθ platform	ROE-200	Sutter Inc.	Manual remote Control the XYZθ platform
10	Other auxiliary equipment	A personal computer, a vibration isolation platform, and a customized base, et al.		

### Experimental results of the automatic microinjection system

#### Preparation for the experimental study

First, we prepared a batch of zebrafish embryos and 2-dfp larvae to be injected. The larvae were adequately anesthetized using 168 mg/L tricaine (MS222) at the beginning of microinjection. Then, we used a micropipette to transfer the anesthetized samples to the MAM and adopted a brush to push the samples into the corresponding grooves. Benefiting from the effect of anesthesia and the specifically designed forward (head up) and backward (head down) arrays, the zebrafish embryos and larvae are quickly placed into the corresponding grooves. After, the MAM containing embryos and larvae is fixed on the XY platform, and the microscope is adjusted to obtain a clear image of only one sample in the field of view (FOV).

Before conducting microinjection, the microneedle is installed on the microinjector, and the microinjector needs to be fixed to the XYZθ platform at a 45° tilt angle relative to the horizontal plane. By adjusting the joystick of the XYZθ platform, the needle tip of the microneedle is positioned at the center of the XY plane and contacts the agarose surface in the *z*-direction. We record the spatial coordinates of the point and set it as injection point *B*. Then, we move the microneedle back 2000 µm in the *x* and *z* directions, record the spatial coordinates of the point, and set it as standby point *A*. This processing technique ensures the capability to realize arbitrary movement of the sample in the horizontal plane and avoid interference from the needle on the visual recognition algorithm. When performing a microinjection task, the needle moves diagonally from standby point *A* to injection point *B* at a 45° angle to complete the puncture action. After the injection is completed, the needle returns to standby point *A*.

**Fig. 5:**
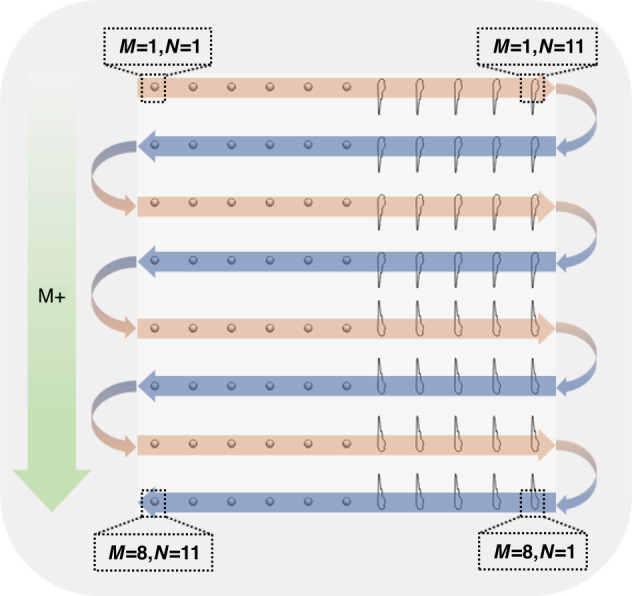
Schematic diagram of the automatic microinjection path

The path planning of batch microinjection is also a significant factor affecting injection efficiency. The schematic diagram of the automatic microinjection path is demonstrated in Fig. 5, where *M* represents the number of rows and *N* represents the number of columns. In this work, the maximum of *M* is 8, and the maximum of *N* is 11. One cycle ideally completes microinjection of 88 samples, including 48 zebrafish embryos and 40 zebrafish larvae. To reduce unnecessary movement of the MAM, after executing a row of microinjections, it will move to the next nearest row and then complete the microinjection task in reverse. In other words, if the sample in the upper-left corner is set to *M* = 1 and *N* = 1, the automatic microinjection will be executed from left to right in odd-numbered rows and from right to left in even-numbered rows. This path planning will further improve the efficiency of the automatic microinjection system.

#### Batch microinjection performance testing

**Fig. 6:**
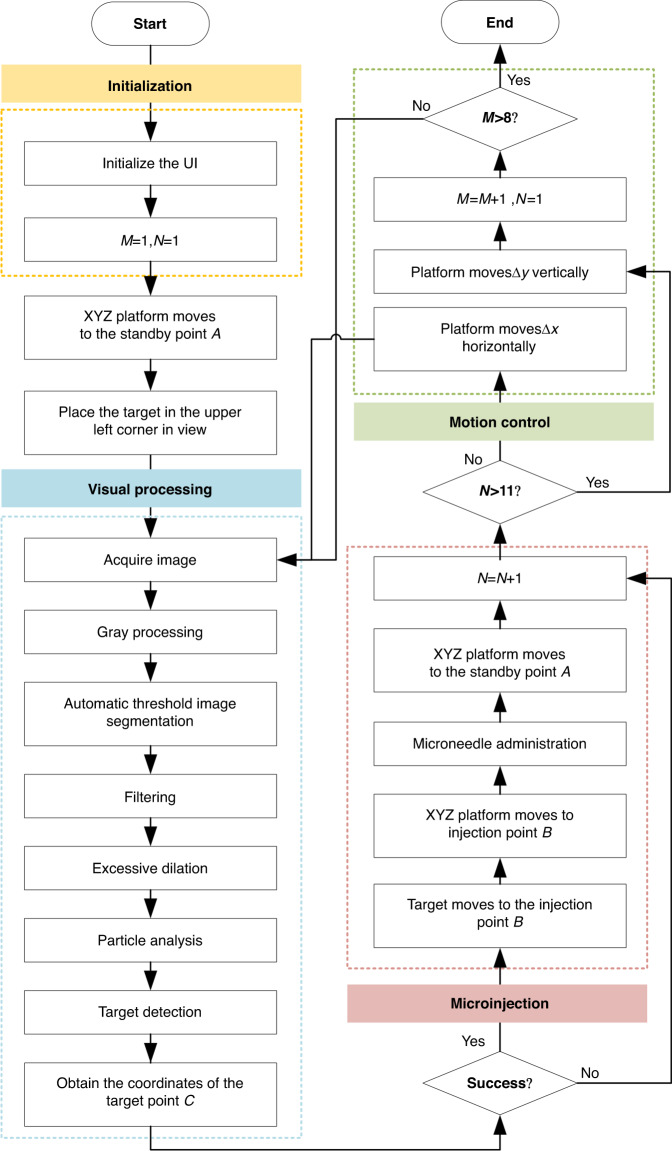
Flowchart of a complete automatic microinjection cycle

Specific experimental studies were conducted to verify the effectiveness and performance of the designed automated microinjection system for batch zebrafish embryos and larvae. The flowchart of a complete automatic microinjection cycle is illustrated in Fig. 6.

After the user clicks the start button, the program will initializes. In this process, the data from the last run in the UI will be cleared, all buttons will be reset, and the values of M and N will be set to 1. Then, the XYZθ platform carrying the microinjector and microneedle will move to standby point *A*. In advance, we adjust the joystick of the XY platform to ensure that the sample in the upper-left corner of the MAM is moved to the center of the FOV.

Then, the system enters the visual processing program and performs the following steps in sequence: obtaining color images, grayscale processing, automatic threshold image segmentation, filtering, excessive dilation, particle analysis, target detection, and obtaining the coordinates of target point *C*. The microinjection program is entered if the embryo or larval yolk center is successfully identified. If the recognition is unsuccessful, the next target recognition is carried out.

During the execution of the microinjection program, the XY platform moves the target object to injection point *B* based on the visual recognition results. Then, the XYZθ platform carries the microneedle to injection point *B* to complete the puncture action. The microinjector will release medication, and after completion, the microneedle returns to standby position *A*. Next, we increment the value of *N*. If *N* ≤ 11, then the microinjection task for a row has not been completed. The XY platform moves horizontally with MAM for a distance of Δ*x*, places the next target in the FOV, and then executes the corresponding program for visual processing and microinjection again. Until *N* is greater than 11, a row of injection tasks is completed, and the XY platform carries the MAM vertically by a distance of Δ*y*. Then, we increment to the value of *M* and reset *N* to 1. When *M* ≤ 8, all microinjection tasks have not been completed, and the corresponding programs for visual processing and microinjection are executed again. When *M* > 8, all microinjection tasks of this cycle have been completed, and the program operation ends

**Fig. 7:**
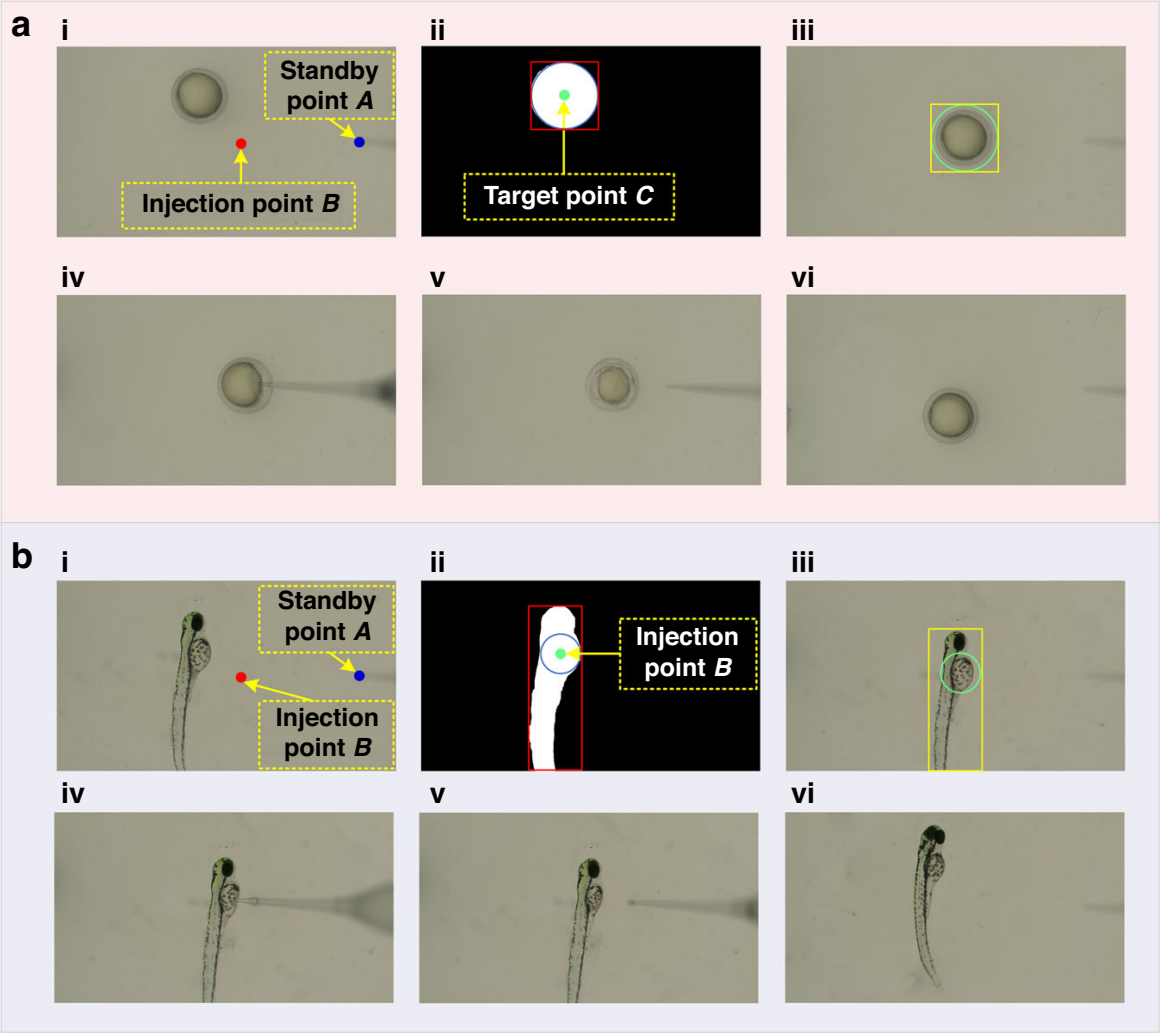
**Automatic microinjection process.** **a** Process of automatic microinjection for one zebrafish embryo. **b** Process of automatic microinjection for one zebrafish larva

Figure 7a, b demonstrates the complete process of automatic microinjection for one zebrafish embryo and one larva. As shown in Fig. 7a-i, the blue dot in the figure represents standby point *A*, and the red dot represents injection point *B*. After the visual recognition program, the coordinate information of target point *C* is obtained and marked as a green dot, as shown in Fig. 7a-ii. After calculating the relative distance from point *C* to point *B*, the XY platform carries the target object and moves it to injection point *B*, as shown in Fig. 7a-iii. Then, the XYZθ platform brings the microneedle from standby point *A* to injection point *B* to complete the puncture action, as shown in Fig. 7a-iv. After completing administration, the microneedle returns to standby point *A* again, as shown in Fig. 7a-v. Finally, we move the next sample to be injected into the center of the FOV, as shown in Fig. 7a-vi. The complete microinjection process of a zebrafish larva is illustrated in Fig. 7b-i to Fig. 7b-vi, and its principle and process are consistent with those of a zebrafish embryo.

To better describe the system performance, we define the following indices. The total number of samples in one injection task is *N*_total_, the number of successful microinjection samples is *N*_suc_, and the number of survival samples on the second day is *N*_sur_. The time needed for the entire microinjection process is *T*_total_. Based on the above definition, the following formulas are derived:

12 $${R}_{{\rm{suc}}}=\frac{{N}_{{\rm{suc}}}}{{N}_{{\rm{total}}}}$$

13 $${R}_{{\rm{sur}}}=\frac{{N}_{{\rm{sur}}}}{{N}_{{\rm{suc}}}}$$

14 $${T}_{{\rm{suc}}}=\frac{{T}_{{\rm{total}}}}{{N}_{{\rm{suc}}}}$$

15 $${T}_{{\rm{sur}}}=\frac{{T}_{{\rm{total}}}}{{N}_{{\rm{sur}}}}$$

where *R*_suc_ is the success rate, *R*_sur_ represents the survival rate, *T*_suc_ is the average successful injection time of a single sample, and *T*_sur_ denotes the average successful injection and continued survival time of a single sample.

An experimental investigation was conducted to compare the different performances of the developed automatic microinjection system versus traditional manual injection. During manual injection, the operator chose the conventional and commonly used microinjection process in biological experiments and completed the microinjection task of 88 samples. The specific operation process used a pipette to place 48 zebrafish embryos and 40 zebrafish larvae into the V-shaped slot of the Petri dish and conduct puncture injection with the help of a microscope. Recording the corresponding *T*_total_ and success numbers *N*_suc_ for every 11 sample microinjection tasks is completed. To ensure a more scientific process on the survival numbers *N*_sur_, we chose to observe all the injected samples on the second day and record the number of survivors. After calculating other performance parameters through Eqs. (12)–(15), all results were obtained. As Table 2 shows, the manual microinjection tool needed an average of 38.30 s to complete a microinjection task. The successful injection rate *R*_suc_ of 88 samples was 85.23%. To reduce artificial injury caused by excessive movement of the injected samples, only the survival rate of all samples after the injection was counted with *R*_sur_ = 89.33% and *T*_sur_ = 42.88 s.

**Table 2:** Experimental results of manual operation

*N*_total_	*T*_total_ (s)	*N*_suc_	*R*_suc_ (%)	*T*_suc_ (s)	*N*_sur_	*R*_sur_ (%)	*T*_sur_ (s)
11	366.08	9	81.82	40.68	-	-	-
22	710.49	19	86.36	37.39	-	-	-
33	1021.02	30	90.91	34.03	-	-	-
44	1348.16	41	93.18	32.88	-	-	-
55	1684.65	51	92.73	33.03	-	-	-
66	2063.27	59	89.39	34.97	-	-	-
77	2452.78	68	88.31	36.07	-	-	-
88	2872.65	75	85.23	38.30	67	89.33	42.88

Then, according to the program flowchart in Fig. 6, we used the developed automatic microinjection system to conduct batch microinjection of 88 samples preplaced on the MAM, including 48 zebrafish embryos and 40 zebrafish larvae. To provide a more comprehensive description of the injection process, we treated each row of 11 samples as a group and recorded the injection time *T*_total_ and success numbers *N*_suc_ for each group. The counting method for survival numbers *N*_sur_ was consistent with manual microinjection. Based on the calculations of Eqs. (12)–(15), all results were determined as listed in Table 3. The data show that as the number of injected samples increases, although the injection success rate *R*_suc_ fluctuates, it remains above 90%. After completing the microinjection of 88 samples, the injection *R*_suc_ reached 92.05%. *T*_suc_ also steadily changed, ultimately reaching 13.88 s. The result of *R*_sur_ was 93.83%, and the *T*_sur_ was 14.79 s.

**Table 3:** Experimental results of the automatic microinjection system

*N*_total_	*T*_total_ (s)	*N*_suc_	*R*_suc_ (%)	*T*_suc_ (s)	*N*_sur_	*R*_sur_ (%)	*T*_sur_ (s)
11	137.83	10	90.91	13.78	-	-	-
22	299.86	20	90.91	14.99	-	-	-
33	444.73	31	93.94	14.35		-	-
44	577.94	41	93.18	14.09	-	-	-
55	705.21	52	94.55	13.56	-	-	-
66	835.56	62	93.94	13.48	-	-	-
77	982.63	72	93.51	13.65	-	-	-
88	1124.42	81	92.05	13.88	76	93.83%	14.79

**Fig. 8:**
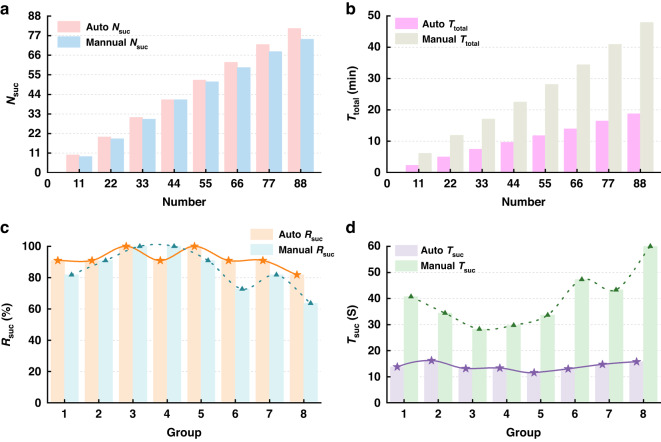
**Experimental results.** **a** Results of *N*_suc_ during automatic and manual injection processes. **b** Results of *T*_total_ during automatic and manual injection processes. **c** Results of *R*_suc_ during automatic and manual injection processes in different groups. **d** Results of *T*_suc_ during automatic and manual injection processes in different groups

Figure 8a more intuitively shows that the *N*_suc_ of automatic microinjection for the same number of samples is generally higher than that of manual microinjection. After completing the injection task of 88 samples, the *N*_suc_ of automatic microinjection increased by 8% compared to manual microinjection. As Fig. 8b shows, automatic microinjection consumes less time than manual microinjection when completing the same number of sample injections. After completing the injection task of 88 samples, automatic microinjection can reduce time consumption by 60.86% compared to manual microinjection. Figure 8c, d illustrate the changes in *T*_suc_ and *R*_suc_, which are obtained through automatic and manual microinjection of 8 groups of samples. As shown in the blue dotted curve in Fig. 8c, the manual injection success rate tends to be low, and after increasing, it showed a downward trend. The low success rate of manual injection initially may be due to the need for a user to familiarize themself with the injection environment. After completing five sets of injection tasks, there was another downward trend, which is likely caused by visual fatigue induced during prolonged work. The success rate of automatic injection remains relatively stable and high. The curves of *T*_suc_ also exhibited the same rhythms. Through calculation, the range of *T*_suc_ for manual injection was 7.80 s, larger than that of automatic injection (1.51 s). This may be due to the susceptibility of manual injection to external environmental interference. In addition, the standard deviation of *T*_sus_ for the two methods was calculated to further evaluate injection stability. The standard deviation of 8 sets of data obtained through automatic microinjection was 1.53242, which was significantly smaller than the results of manual microinjection (10.54446). This result indicates that the developed microinjection system also exhibits high stability, further indicating that the system can continuously provide reliable microinjection operation with high throughput.

## Discussion

Table 4 summarizes the main performances (*T*_suc_, *R*_suc_, and *R*_sur_) of the current microinjection system for different injection targets of zebrafish. Previously demonstrated tools can inject only a single type of target (e.g., zebrafish embryos or larvae), whereas the automatic microinjection system developed in this work can support batch microinjection of these two typical samples simultaneously. The proposed system can achieve a success rate of 92.05% and a survival rate of 93.83%, and the time needed to inject a single sample successfully is only 13.88 s, demonstrating its advantages of versatility and excellent performance.

**Table 4:** Performance comparison versus existing work

References	Injection target	*T*_suc_	*R*_suc_	*R*_sur_
^33^	Embryos	4 s	99%	98%
^34^	Embryos	4 s	100%	98%
^44^	Embryos	3.55 s	-	46%
^42^	Larvae	79.79 s	94%	100%
^48^	Larvae	47.06 s	85%	94.1%
^49^	Larvae	46.81 s	94%	100%
^50^	Larvae	18.61 s	90%	96.3%
This work	Embryos and larvae	13.88 s	92.05%	93.83%

## Conclusion

In conclusion, a novel robot microinjection system dedicated to batch injection of both zebrafish embryos and larvae has been developed. The system includes a vision recognition algorithm based on an automatic threshold and excessive dilation to simultaneously identify the center of the zebrafish embryo and the center of the larval yolk. In addition, specialized operating software is built with a program framework that is based on an efficient producer-consumer model and a user-friendly UI that is designed to facilitate operation. Moreover, a novel microstructural agarose medium is designed and manufactured to immobilize batches of zebrafish embryos and larvae simultaneously, and a prototype system consisting of multiple hardware devices is developed. In addition, a novel and efficient automatic microinjection workflow is proposed to realize high-throughput microinjection. Finally, experimental tests are conducted to assess the performance of the developed system. Through microinjection of a batch of zebrafish embryos and larvae, the injection success rate reached 92.05%, the survival rate was 93.83%, and the single successful injection time was only 13.88 s, values that are significantly better than those recorded for manual injections. These results reveal that our system can provide automatic microinjection of zebrafish embryos and larvae with excellent efficiency, a high success rate, a high survival rate, and sufficient stability. Due to the aforementioned advantages, the automatic microinjection system holds significant potential for application in biological and pharmaceutical research. It facilitates the timely injection of materials into batches of zebrafish embryos and larvae, particularly in the context of large-scale screening of biomolecules or drug compounds. In future research, a force feedback system should be added to reduce the damage caused by puncture force to samples and further improve the survival rate during the injection process. Moreover, there is potential for the appropriate improvement of the system to extend its application to other model organisms, such as Drosophila embryos, mouse oocytes, and *C. elegans*.

## Materials and methods

Due to its hydrophilicity and absence of charged groups, agarose is not easily denatured and adsorbed on sensitive biological macromolecules, making it an ideal inert carrier^21^. To ensure structural stability and avoid damage to zebrafish caused by relatively hard agarose, a double-layer design is adopted to make the MAM. The lower layer uses high-concentration agarose to make a relatively hard base. The upper layer was inverted with low-concentration agarose to create microarray grooves.

Specifically, we first mixed agarose powder with Sigma solution at a concentration of 2% and then heated it at 90–95 °C for 2 min. After continuous stirring and cooling, we poured it into a Petri dish and allowed it to stand for 30 min until it solidified to form a relatively hard lower layer. Then, we prepared an agarose solution with a concentration of 1% to make the top layer. Similarly, we heated it at 90–95 °C for 2 min, stirred it constantly, and then poured it into a Petri dish containing the lower layer after it cooled. At this time, we slowly inverted the 3D-printed mold into the Petri dish and attempted not to leave bubbles. We allowed it to stand for another 30 min until the upper layer solidified and then removed the 3D-printed mold. Hence, the MAM with batch grooves for immobilizing zebrafish embryos and larvae was completed, as shown in Fig. 4a.

### Supplementary information

Supplementary Video
